# A strategy of novel molecular hydrogen-producing antioxidative auxiliary system improves virus production in cell bioreactor

**DOI:** 10.1038/s41598-024-54847-7

**Published:** 2024-02-19

**Authors:** Yu-Jing Zeng, Min-Kung Hsu, Jia-Rong Cai, Hsian-Yu Wang

**Affiliations:** 1 International Degree Program in Animal Vaccine Technology, International College, National Pingtung University of Science and Technology, Pingtung, 91201 Taiwan; 2 Graduate Institute of Animal Vaccine Technology, College of Veterinary Medicine, National Pingtung University of Science and Technology, Pingtung, 91201 Taiwan; 3 General Research Service Center, National Pingtung University of Science and Technology, Pingtung, 91201 Taiwan; 4 Animal Biologics Pilot Production Center, National Pingtung University of Science and Technology, Pingtung, 91201 Taiwan; 5 Innovative Bioproducts Technical Service Center, National Pingtung University of Science and Technology, Pingtung, 91201 Taiwan

**Keywords:** Antioxidative auxiliary system (AAS), High-density cell bioreactor, Hydrogen molecules (H_2_), Cellular stress response, Viral production, Vaccine, Microbiology techniques, Antimicrobials, Cell growth, Senescence, Biomedical engineering, Biotechnology

## Abstract

In the increasing demand for virus vaccines, large-scale production of safe, efficient, and economical viral antigens has become a significant challenge. High-cell-density manufacturing processes are the most commonly used to produce vaccine antigens and protein drugs. However, the cellular stress response in large-scale cell culture may directly affect host cell growth and metabolism, reducing antigen production and increasing production costs. This study provided a novel strategy of the antioxidant auxiliary system (AAS) to supply molecular hydrogen (H_2_) into the cell culture media via proton exchange membrane (PEM) electrolysis. Integrated with a high-density cell bioreactor, the AAS aims to alleviate cellular stress response and increase viral vaccine production. In the results, the AAS stably maintained H_2_ concentration in media even in the high-air exposure tiding cell bioreactor. H_2_ treatment was shown safe to cell culture and effectively alleviated oxidative stress. In two established virus cultures models, bovine epidemic fever virus (BEFV) and porcine circovirus virus type 2 (PCV-2), were employed to verify the efficacy of AAS. The virus yield was increased by 3.7 and 2.5 folds in BEFV and PCV-2 respectively. In conclusion, the AAS-connected bioreactor effectively alleviated cellular oxidative stress and enhanced virus production in high-density cell culture.

## Introduction

Bioreactors play an important role in producing large-scale vaccines in cell culture, which can provide more efficient and cost-effective vaccine production. Large-scale and high-density conditions in bioreactors may cause cellular stress response and then decrease viral production^1–3^. Many cellular stress responses, such as cellular oxidative stress, ER stress, and senescence-like response, could inhibit or decrease virus amplification in the cell culture system. In previous research, the cellular oxidative response has been reported to decrease Chinese Hamster Ovary (CHO) cell-producing protein drugs^4,5^. In our previous study, the stress-induced senescence-like response also inhibited BEFV virus replication in the BHK-21 cells^6^. Thus, how to resolve cellular stress response in high-density cultivation still was a formidable challenge in bioreactors.

Oxidative stress refers to an imbalance between free radical production and intracellular antioxidant responses in a cell^7–9^. When reactive oxygen species (ROS) are excessively produced, they may trigger lipid peroxidation, protein denaturation, and nucleic acid damage. Therefore, oxidative stress causes severe damage to cells in vivo and in vitro environments^10,11^. ROS production can trigger the host cells' antiviral inflammatory response in viral infections^12^. Consequently, regulating ROS levels to maintain cellular homeostasis in a bioreactor was very important to the biopharmaceutical industry. The cellular oxidative stress in the manufacturing process may arise in conditions including uneven oxygen distribution, high cell density, lack of nutrients, waste accumulation, and virus infection. The common methods of eliminating ROS production are reducing mechanical damage or directly adding antioxidant chemical reagents in the bioreactor, such as vitamin C, vitamin E, and glutathione^13,14^. However, direct additional chemical antioxidants in the culture media not only require further isolation process and monitoring residuals but also difficult to quickly adjust their amounts with the manufacturing process. Here, a new strategy by using H_2_ as the antioxidant material to reduce oxidative stress response quickly, easily, and with no residuals in the production process.

H_2_ is a novel antioxidant material to efficiently reduces oxidative stress^15,16^. The water molecules dissociate weakly, splitting into H^+^ and OH^−^ ions. Through electrolysis, under the influence of electron reception at the cathode, hydrogen ions transform into H_2_ highly efficiently. The biological activity of H_2_ and hydrogen-rich water has been extensively studied in humans, animal models, and cell cultures. Many previous studies reported that H_2_ can quickly reduce the oxidants in human fibroblast cells induced by irradiation damage^17–19^.

Here, we designed an antioxidant device, the antioxidative auxiliary system (AAS), to directly generate H_2_ within cell culture media in a high-density bioreactor. The results showed that the AAS supplied H_2_ into the cell culture medium to stabilize cellular oxidative stress and increase virus yields in the bioreactor. Two virus culturing models, BEFV in BHK-21 cells and PCV-2 in PK-15 cells were employed. In the virus production processes, the AAS device effectively reduced oxidative stress and increased virus yields by 2 to 4 folds. The present research is the first to apply H_2_ for viral vaccine production.

## Results

### Device design for AAS connected with BelloCell-500AP lab-scale bioreactor

**Figure 1:**
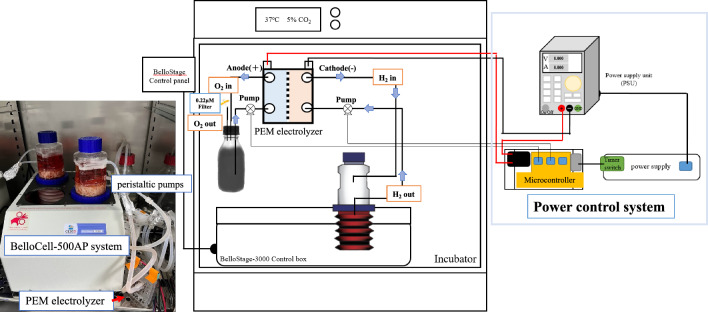
The layout of AAS connection with a lab-scale bioreactor BelloCell-500AP.

The AAS system included a power-controlling unit and a PEM electrolysis core. The power control unit was constructed with a microcontroller, power supply, and timer-connected power, which was used to achieve different requirements such as current, voltage, and activation times. The PEM electrolysis core is centered around connecting the BelloCell-500AP system (cathode), a pure water system (anode), and pumping by two peristaltic pumps (Fig. 1). H_2_ was electrolyzed directly into the culture media by the PEM electrolysis core in the cathode, and the O_2_ was generated in the anode in pure water. In the cathodic region of water electrolysis, the reduction of water molecules generates hydroxide ions (OH^−^), resulting in an alkaline environment of the culture media flowing through the cathode and could be used to balance the cell-secreted acid during growth. A 0.22 µm filter was provided to the pure water system to vent the O_2_ gas out. In the BelloCell-500AP lab-scale bioreactor, H_2_ will gradually dissipate through the BelloCell-500AP air venting filter. Thus, the power control system was needed to regulate proper activation times and maintain H_2_ concentration in the culture environment. The advantage of this design is that it could be adjusted for various experimental parameters, including the activation frequency of the electrolysis core, voltage, current, and the flow rate of the supplementary buffer.

**Figure 2:**
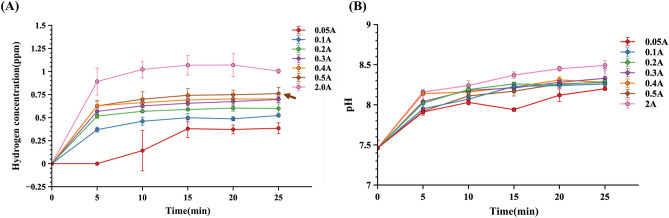
Higher current values could produce more H_2_ concentration and higher pH by AAS in a 500 ml MEM culture medium without cells with 15-min activation and 15-min deactivation intervals. (**A**) H_2_ concentration was detected by Dissolved Hydrogen Meter-DH30. (**B**) The pH values were monitored by the LAQUAact pH-22 pH meter. The brown arrow indicates the current of 0.5 A described in the “Result” section.

First, the device was tested by using different current values (0.05 A, 0.1 A, 0.2 A, 0.3 A, 0.4 A, 0.5 A, 2 A) with 15-min activation and 15-min deactivation intervals in MEM medium without cells for the H_2_ generation estimation. The results demonstrated that H_2_ concentrations could achieve 0.6–0.8 ppm within 25 min in a 500 ml culture medium by a current of more than 0.2 A, and could achieve 1 ppm with a current of 2 A (Fig. 2A). However, with the increase in current and time, the pH value of the MEM medium increased rapidly (Fig. 2B). However, since high pH will hurt cells, the current must be regulated carefully.

Actually, using a high current, 2 A, can achieve higher hydrogen concentrations, but excessive pH and bubble formation during the electrolysis process will be harmful to the cells. A constant 0.5 A condition was employed for all experiments to make 0.6 ~ 0.8 ppm in the lab-scale BelloCell-500AP bioreactor. The H_2_ concentration and pH within the bioreactor can be regulated quickly. This feature allows AAS to be adjusted for different bioreactors easily.

### The AAS showed no cytotoxicity and reduced cellular oxidative stress in the BelloCell-500AP

**Figure 3:**
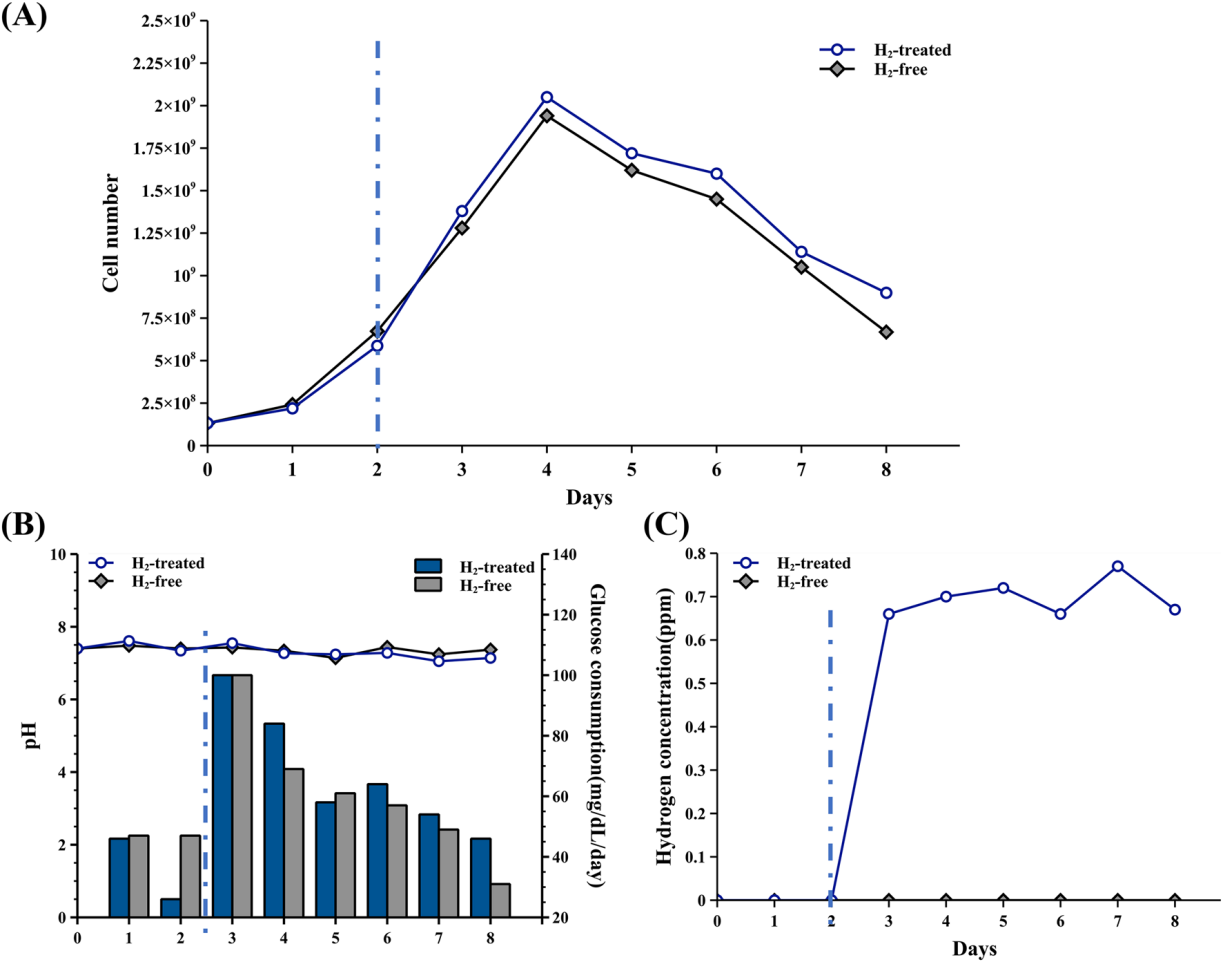
The cell growth curve and glucose consumption rate were similar between BHK-21 in the AAS-connected BelloCell-500AP and the control bottle. (**A**) The cell number of H_2_-treated and H_2_-free groups was monitored every day. (**B**) The glucose concentration was analyzed and calculated into daily glucose consumption (mg/dl/day). The pH level was also monitored every day. (**C**) The H_2_ concentration in the culture medium was analyzed by Dissolved Hydrogen Meter-DH30. The blue dashed line divides the figure into hydrogen-free (left side) and AAS-started hydrogen-positive (right side) culture periods.

To confirm whether the cells showed cytotoxicity or growth inhibition by using the AAS-linked bioreactor, the cell number and glucose-consuming rate were employed as indicators. After BHK-21 cells were inoculated on Day 0, the matrix from the AAS-linked or the control bioreactor was sampled for cell number calculation every day from Day 1 to Day 8. A fed-batch method was employed in this experiment. The growth media was exchanged only once on day 2. From day 3 to day 8, glucose was exclusively supplied at a final concentration of 100 mg/dl in the existing growth media. The cell populations reached maximum growth on day 4, followed by a period of decreasing cell numbers due to the sole supply of glucose. On day 8, the final day, only a sparse population of cells remained in the bioreactor. This process facilitated the establishment of conditions characterized by cell growth increment and starvation decrement within a single culture period, allowing for easy comparison of the two growth conditions. In Fig. 3A, cell growth curve results, both the H_2_-treated and H_2_-free groups reached the peak of cell counts on day 4, with values of 2.0 x 10^9^ cells/bt and 1.9 x 10^9^ cells/bt, respectively. The cell number started to shrink after day 4 both in H_2_-treated or H_2_-free groups. The results indicated that AAS-generated H_2_ treatment did not cause cytotoxic effects in growing and stressing conditions. The glucose consumption rate also showed similar results, the H_2_-treated group did not significantly decrease cellular glucose metabolism. Furthermore, the pH remained consistently within the range of 7.4 to 7.6 during the entire culture period, this indicates that the PEM electrolyzed will not cause pH quick raising in this experiment condition (Fig. 3B). The dissolved hydrogen concentration is analyzed to evaluate the H_2_-supplying efficiency of the AAS when connecting BelloCell-500AP. The results showed that hydrogen concentration in the bioreactor could be maintained between 0.6-0.8 ppm stably in the conditions of AAS 15 minutes start, 15 minutes stop, and 0.5 A current (Fig. 3C).

In order to verify the antioxidant efficacy of the AAS system in high-density cell culture, BHK-21 cells were inoculated into BelloCell-500AP and cultured as described in the previous section. The cells were trypsinized from the matrix on day 4, day 5, and day 6 for the ROS generation assay. After day 4, the cells faced starvation-stressing conditions without growth media exchange. In the ROS assay, the cellular oxidative stress was induced by H_2_O_2_ as the indicated concentration followed by flow cytometry analysis to evaluate the ROS tolerance response in the cells treated by AAS-generated H_2_ and in the H_2_-free control group.

**Figure 4:**
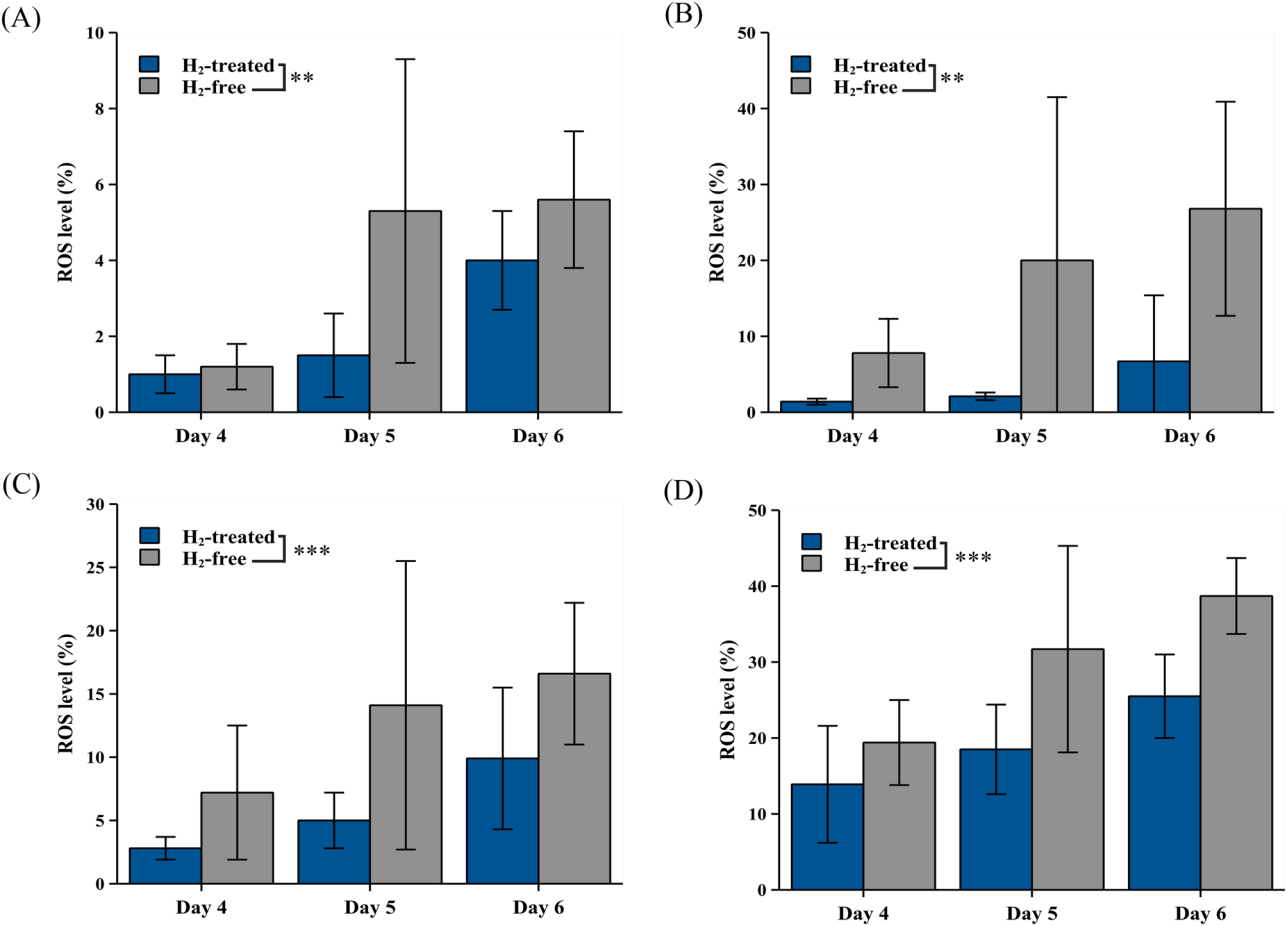
The AAS reduced cellular oxidative stress in the BelloCell-500AP bioreactor. The BHK-21 cells were isolated from day 4 to day 6 and were induced with (**A**) 0 μM H_2_O_2_, (**B**) 4 µM H_2_O_2_, (**C**) 10 µM H_2_O_2_, and (**D**) 20 μM H_2_O_2_ followed by ROS levels analysis by flow cytometry. Results represent the mean ± standard deviation (SD) in three individual cell sample repeats. The data were analyzed by two-way ANOVA. Tukey’s HSD tests analyzed further multiple comparisons. The significance between the two group cells was accepted at p < 0.01** and p < 0.001***.

The results showed that the cellular ROS levels increased along with the culture time, but the ROS levels in H_2_-treated cells were significantly lower than in the H_2_-free group (Fig. 4A). The difference between H_2_-treated and H_2_-free groups more clear in 4 µM, 10 µM, and 20 µM H_2_O_2_ induction (Fig. 4B–D). In conclusion, the AAS could effectively alleviate cellular oxidative stress in the high-density cell bioreactor.

### The AAS-connected BelloCell-500AP improves BEFV production in BHK-21 cells

**Figure 5:**
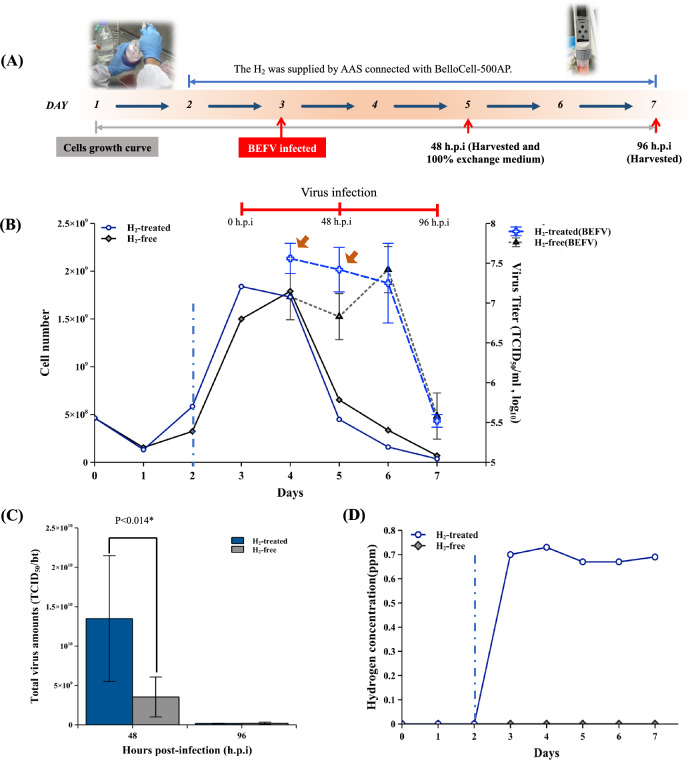
The BEFV production was improved by AAS in BelloCell-500AP. (**A**) The BEFV infected BHK-21 cells production process with AAS connected BelloCell-500AP. (**B**) Cell growth curve and virus titer. (C) All virus culture media was exchanged by new media at 48 and 96 h.p.i. The total virus amounts in the bulks were analyzed. (**D**) Hydrogen concentration was analyzed every day. The blue dashed line divides the figure into hydrogen-free (left side) and hydrogen-positive (right side) culture periods. The brown arrow indicates the quick virus increment described in the result section. Student’s t-test performed the indicated statistical analysis. The significance was accepted at p < 0.05*.

The BEFV virus replicates in BHK-21 cells rapidly and causes obvious cytopathic effects (CPE) within 48 h. Here, the BEFV model was used to estimate the effects of the AAS-connected bioreactor for quick virus growth in high-density cell conditions. The experimental process is shown in Fig. 5A. BHK-21 cells were inoculated into two BelloCell-500AP bottles at 4.6 × 10^8^ cells/bt, and the cell number reached 1.5 and 1.8 × 10^9^ cells/bt on day 3, then were inoculated with BEFV, and the virus titer from 24 to 96 h post-infection (h.p.i) were analyzed. The AAS was turned on 24 h before virus inoculation in the H_2_-treated group (from day 2 to day 7).

The results showed that the cells were consumed by the virus very quickly in H_2_-treated groups at 24- and 48-h.p.i. The virus titer in the H_2_-treated group increased faster than in the H_2_-free group (Fig. 5B brown arrows). After 48 h.p.i, only rare cell residual in the bioreactor, and the virus titer shows low at 96 h.p.i. The total amount of virus antigens in the harvest bulks at 48 and 96 h.p.i are shown in Fig. 5C. The total virus harvest in H_2_-treated group was significantly higher than in H_2_-free control group at 48 h.p.i. The fold change of H_2_-treated over H_2_-free groups at 48 and 96 h.p.i are 3.96 and 0.87 as indicated (Supplementary Fig. S1). Since the BEFV usually causes the CPE close to 72 h.p.i, the virus titer in the H_2_-free group showed a quick rise at 72 h.p.i (Fig. 5B day 6), like the general condition. However, the total amount of virus yield harvested in the H_2_-treated group was significantly higher than the H_2_-free group (3.7-fold) (Supplementary Table 1). In this virus culture experiment, the AAS can stably supply dissolved H_2_ as 0.6–0.8 ppm during virus infection (Fig. 5D).

### The AAS-connected BelloCell-500AP improves PCV-2 production in PK-15 cells

**Figure 6:**
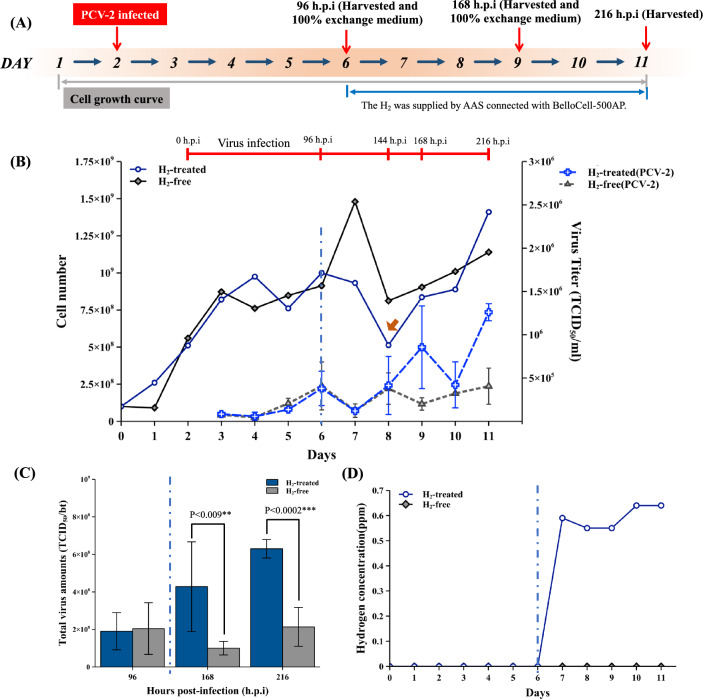
The PCV-2 production was improved by AAS in BelloCell-500AP. (**A**) The PCV-2 infected PK-15 cells production process with AAS connected BelloCell-500AP. (**B**) Cell growth curve and virus titer. (**C**) All virus culture media was exchanged by new media at 96, 168, and 216 h.p.i. The total virus amounts in the bulks were analyzed. (**D**) Hydrogen concentration was analyzed every day. The blue dashed line divides the figure into hydrogen-free (left side) and hydrogen-positive (right side) culture periods. Student’s t-test performed the indicated statistical analysis. The significance was accepted at p < 0.05*, p < 0.01**, and p < 0.001***.

In PCV-2 production tests, the PCV-2 infects PK-15 cells in low-density conditions (1.04 × 10^8^ cells/bt) at the beginning and the virus replication rate is slow. This lets us harvest the virus antigen several times in one bioreactor. The experimental process is demonstrated in Fig. 6A, the AAS was turned on at 96 h.p.i to minimize the cellular stress response induced by the following high cell density and virus replication condition.

Before activating AAS, the PK-15 cell numbers reached 7.6 × 10^8^ cells/bt and 8.5 × 10^8^ cells/bt in the H_2_-treated group and H_2_-free group on 96 h.p.i, respectively. Subsequently, the PK-15 cells decreased quickly during 96 ~ 144 h.p.i, especially in the H_2_-treated group. In this period, the virus titer in the H2-treated group increased more rapidly than in the H2-free group. Between 144 ~ 216 h.p.i, cell numbers in both groups gradually recovered, with the H2-treated group exhibiting faster growth, and the virus titer being higher than in the H2-free group (Fig. 6B). This virus growth may have resulted from the PCV2 genomic DNA being dependent on cell host replication. The culture medium was exchanged at 96 h.p.i (day 6) and 168 h.p.i (day 9). Consequently, cell growth was observed on days 7 and 10. The cell death peak and lower cell numbers were observed on day 8. Similarly, the virus supernatant was harvested on day 9 by replacing fresh culture media, resulting in a lower virus titer on day 10. Notably, the PCV2 virus titer may not exhibit a 100% correlation with cell death. We observed at least two waves of PCV-2 replications, with the first wave occurring at 144 h.p.i (Fig. 6B, brown arrow) and the second wave extending beyond 216 h.p.i. In Fig. 6B, the results show the dynamic changes in cell number and virus titer in the cell bioreactor.

The amount of virus antigens in the harvest bulks at 96, 168, and 216 h.p.i are shown in Fig. 6C. Before turning on AAS (at 96 h.p.i), the amounts of virus yield in the two groups were similar, but the virus in the H_2_-tread group was significantly higher than in the H_2_-free control group after AAS linked in (168 and 216 h.p.i) (Fig. 6C). The H_2_-treated process enhanced virus production 4.3-fold at 168 h.p.i and 3.2-fold at 216 h.p.i (Supplementary Fig. S1). The total amount of virus yield harvested in the H_2_-treated group was significantly higher than the H_2_-free group (2.5-fold) (Supplementary Table 1) In this virus culture experiment, the AAS can stably supply dissolved H_2_ as 0.6–0.8 ppm during virus infection (Fig. 6D).

### The AAS alleviated the cellular oxidative stress during virus infection

**Figure 7:**
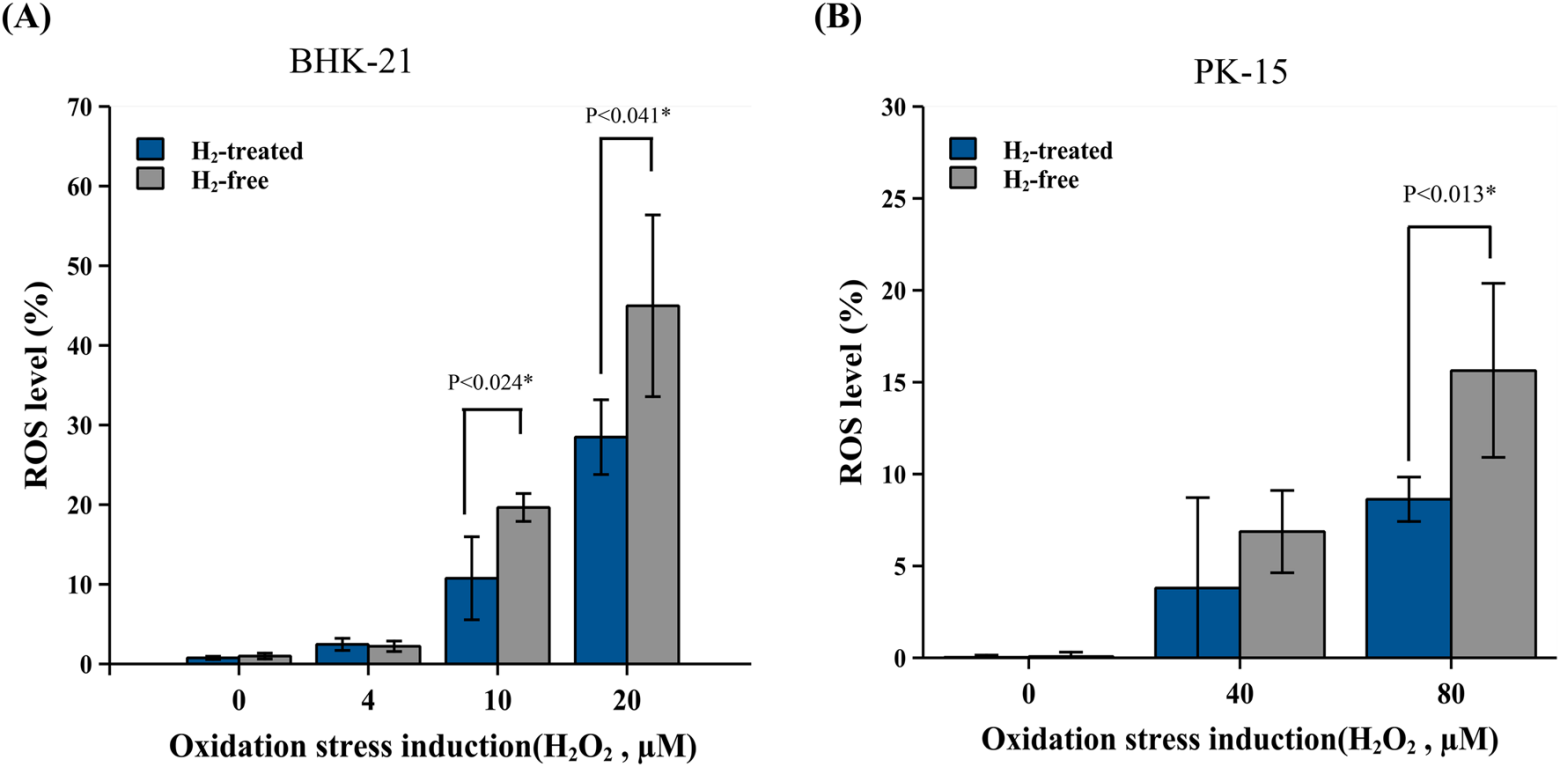
The AAS alleviated the cellular oxidative stress during virus infection at 24 h.p.i. in the BEFV-infected BHK-21 and PCV-2-infected PK-15 models. (**A**) BHK-21 cells infected by BEFV for 24 h and (**B**) PK-15 cells infected by PCV-2 for 24 h were isolated from the bioreactor matrix and induced with different concentrations of H_2_O_2,_ then stained for flow cytometry analysis. Results represent the mean ± standard deviation (SD) in three individual cell sample repeats. A two-tailed Student’s t-test was performed for statistical analysis. The significance was accepted at p < 0.05*.

In order to confirm the antioxidant effect of AAS in the viral infected cells. The cellular oxidative condition was analyzed at 24 h after virus inoculation in BEFV and PCV-2 infection models. The results showed that the virus-induced cellular oxidative stress response was alleviated by using AAS-generated H_2_ treatment in both BEFV (Fig. 7A) and PCV-2 (Fig. 7B). As the higher concentration of H_2_O_2_ induction was used, 10 µM and 20 µM in BEFV and 80 µM in PCV-2, the antioxidant phenomenon became significant.

## Discussion

Maintaining homeostasis in the cell cultured within a high-density bioreactor is a crucial aspect of the biomanufacturing process. Previous research has mentioned that alleviated oxidative stress in bioreactors is advantageous for high-yield virus and recombinant protein production^20,21^. Furthermore, our prior studies also found that stress-induced cellular senescence-like response may inhibit BEFV virus production^6^. How to alleviate the cellular response is important in the pharmaceutical industry when using cell bioreactors. In the present study, the virus production was significantly accelerated by using AAS to alleviate ROS-induced cellular stress response during virus infection.

Cellular stress responses in generally inhibit virus growth and replication through various factors, such as cell inflammation, oxidative stress, cell cycle arrest, and cellular senescence^22–24^. However, Zhang and colleagues also found that PCV-2 benefits from upregulating ROS for virus replication^25–28^. The increment of cellular ROS could induce the nuclear high mobility group box 1 protein (HMGB1) to translocate to the cytosol and enhance the PCV-2 replication in nuclear. The HMGB1 is a ubiquitously present DNA-binding protein that enhances the inflammatory response as a pro-inflammatory cytokine in virus-infected cells^25,29,30^. Some studies have shown that H_2_ negatively regulates HMGB1 function, effectively reducing cell inflammation and apoptosis^31–33^. Therefore, we speculate that AAS-enhanced PCV-2 production not only inhibits the ROS-induced cellular stress response but also negatively regulates HMGB1 to increase PCV-2 production. Furthermore, in Fig. 6, the PK-15 cells continued to grow and produced the virus antigen after 144 h.p.i (day 8). This phenomenon is similar to the virus-persistent cells that were observed in our previous study following BEFV infection in BHK-21 cells^6^. These persistent cells may be caused by cellular stress responses and cellular senescence-like responses, as a cellular antiviral mechanism.

Many studies suggest that molecular hydrogen is a novel and promising antioxidant. However, the limited solubility (approximately 0.00016 g/100 mL in water)^34^ is challenged for its antioxidant effectiveness. The AAS in the present study can stably regulate the flow rate and activation frequency, achieving a consistent supply of molecular hydrogen. This demonstrates that applying an antioxidant device in a bioreactor can provide a long-term, stable supply of H_2_, thereby achieving optimal antioxidant efficiency in the bioreactor system. Directly adding chemical reagents into the cell culture medium may be a convenient way to achieve antioxidant purposes. Nevertheless, using the AAS directly generates H_2_ into the culture media without chemical residual concern would be easier to employ by the GMP manufacturing process.

In the present study, the features of AAS employed in the bioreactor were checked, including (1) the AAS can continuously deliver H_2_ into the culture medium without exhibiting cytotoxicity; (2) the AAS can significantly reduce cellular ROS in the high-density bioreactor; (3) incorporate AAS with bioreactor can increase the BEFV and PCV-2 virus production. In conclusion, we described a novel strategy by using the AAS-connected bioreactor, which effectively alleviated the cellular oxidative stress in high-density cell culture and increased BEFV and PCV-2 production. Using the AAS can be employed by a GMP manufacturing process easily to improve production efficacy.

## Material and methods

### Cell lines, virus strains, and medium

Baby Hamster Kidney BHK-21 cell line (BHK-21) was purchased from the Institute of Bio-resource Collection and Research Center (BCRC), Hsinchu, Taiwan. Porcine Kidney-15 (PK-15) was a kindly gift from Dr. Chun-Yen Chu. Both cell lines were cultured in Eagles Minimum Essential Medium (MEM, Invitrogen, NY, USA) supplemented with 10% fetal bovine serum (FBS, Gibco, Mexico, USA) at 37 °C in an atmosphere of 5% CO_2_. The BEFV strain Tn88128^35,36^ and PCV-2 isolated in Taiwan were adapted in BHK-21 and PK-15 cells respectively. MEM supplemented with 2% FBS is employed for virus culture.

### Lab-scale bioreactor BelloCell-500AP and the connection of antioxidative auxiliary system (AAS)

The BelloCell-500AP lab-scale bioreactor (Esco Bioengineer Co., Taichung, Taiwan) was used as the experimental platform in the present study. The antioxidant auxiliary system (AAS) includes a proton exchange membrane electrolyzer (PEM, NafionTM, DuPont, USA), a single-board microcontroller, two 3D printed peristaltic pumps, and the buffer supplementary. The BelloCell-500AP bioreactor perfusion circulation tube was connected to the cathode of the proton exchange membrane electrolyzer. This can generate H_2_ into cell culture media directly. At the same time, pure water was supplied to the anode. The AAS administered a constant current of 0.5 A and was programmed to activate at 15-minute intervals, followed by a 15-minute rest in periods. This precise regimen was employed to maintain a stable concentration of hydrogen molecules within the culture medium and didn’t change the pH of the BelloCell 500-AP bioreactor during the high-density cell culture period. BelloCell 500-AP stage settings are listed as the following: during cell attachment rising rate 2 mm/sec., top holding time 20 sec., down rate 2 mm/sec. and bottom holding time 0 sec.; during cell culture rising rate 1.5 mm/sec., top holding time 20 sec., down rate 1.5 mm/sec., bottom holding time 20 sec.; during virus infection rising rate of 2 mm/sec., top holding time 30 sec., down rate of 2 mm/sec., bottom holding time of 0 sec. The cell numbers were counted by CVD Nucleus Count Kit (Esco Bioengineer Co., Taichung, Taiwan). The H_2_ and pH of the cell culture media were determined by a Dissolved Hydrogen Meter-DH30 (DH30, Twinno, Taipei, Taiwan) and a LAQUAact pH-22 pH meter (HORIBA Advanced Techno, Co., Ltd., Kyoto, Japan) respectively. The hydrogen concentration will be controlled as 600 ppb-800 ppb upon this condition setting. The glucose concentrations were analysis by the GIucCeII^®^ Glucose Monitoring System (Esco Bioengineer Co., Taichung, Taiwan). Glucose consumption was calculated as the difference between previous glucose concentration and post-glucose concentration according to the equation:

$${\text{Glucose Consumption }} = \, \left( {{\text{Previous Glucose Concentration }} - {\text{ Post Glucose Concentration}}} \right)/{\text{day}}{.}$$

### The measurement of ROS generation

The BioNOC II carrier strips were taken daily from the BelloCell-500AP and were treated with 0.05% trypsin at 10 min then suspended the cells in growth media. After treating the cells with the indicated concentration of H_2_O_2_ at 25 °C avoid light for one hour, the cell reactive oxygen species (ROS) was labeled by the Reactive Oxygen Species (ROS) Detection Assay Kit (ab287839, Abcam, Cambridge, UK) and followed by flow cytometry analysis. The labeling process followed the kit indication. Gently pipette cells up and down to ensure single-cell suspension and analyze on a flow cytometer (BD Accuri™ C6, BD Biosciences, USA). The ROS stain positive cells were showed as percentage within 10,000 cells.

### Virus tittering

The virus samples were centrifuged at 2000 × *g* to separate cell debris. The BEFV amounts were determined by tissue culture infectious dose-50 method, calculated as the Reed and Muench method (1938) and expressed as TCID_50_/mL. The viruses were serially tenfold diluted and applied to wells containing 2 × 10^4^ BHK-21 cells/well in 96-well micro-titration plates (Thermo Scientific Nunc, USA). The plates were incubated at 37 °C, 5% CO_2_ for three days then observed daily for cytopathic effect (CPE). The PCV-2 amounts were determined by qPCR method with a tittered virus sample standard each time and presented as TCID_50_/mL. The PCV-2 viral DNA was isolated by Viral Nucleic Acid Extraction Kit II (GeneAid, Taiwan). The qPCR reactions were performed using QuantiFast SYBR Green PCR Master Mix (Qiagen, Hilden, Germany). The amplification reactions were performed as follows: 95 °C for 3 min and 40 cycles of 95 °C for 15 s., 53.8 °C for 30 s., and 72 °C for 15 s. The primer pairs used for qPCR were PCV-2 detection-F: 5’-AACCACAGTCAGAACGCCC-3’ and PCV-2 detection-R: 5’-AGAAGGGCT GGGTAATGGTG-3’.

### Statistical analysis

Experimental results represent the mean ± standard deviation (SD). The data were analyzed in R software with an agricolae package. The indicated statistical analysis was performed by two-tailed Student’s t-test, or two-way analysis of variance (ANOVA). Further multiple comparisons were analyzed by Tukey’s HSD tests. The significance was accepted at p < 0.05.

### Supplementary Information

Supplementary Figure S1.

Supplementary Table 1.

## Data Availability

The datasets used and analyzed during the current study are available from the corresponding author on reasonable request.
