# Supplementary material for: A strategy of novel molecular hydrogen-producing antioxidative auxiliary system improves virus production in cell bioreactor
Source: Sci Rep. 2024 Feb 19;14:4092. doi: 10.1038/s41598-024-54847-7 (PMC10876984; doi:10.1038/s41598-024-54847-7)
Supplement: Supplementary file 1 — Supplementary Figure S1. [file 41598_2024_54847_MOESM1_ESM.pdf]

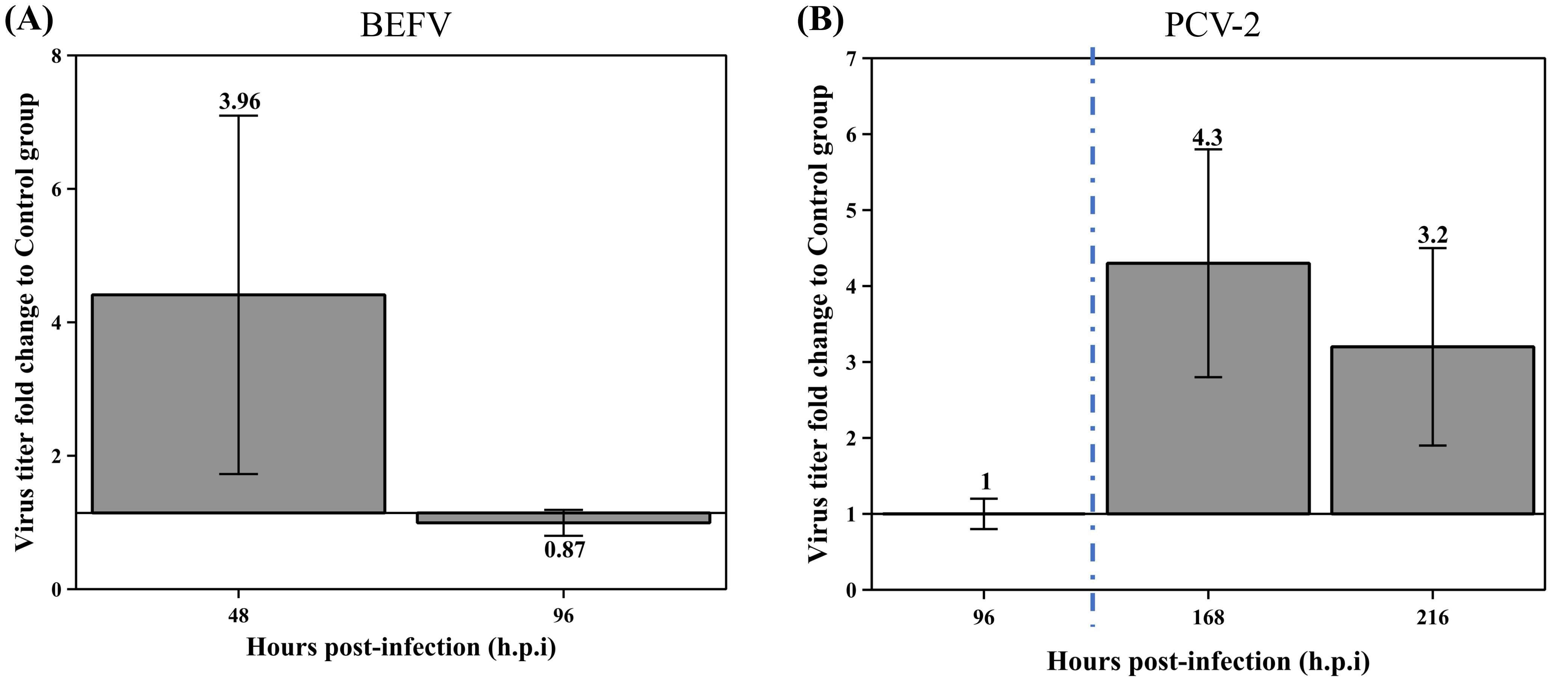

Supplementary Figure 1. The virus fold-change results of AAS treated culture group. The virus yields of the BEFV and PCV-2 were improved by AAS when connected to BelloCell-500AP. (A) The BEFV antigen production yields harvested at 48 and 96 h.p.i were normalized to the H_2-free control group. (B)The PCV-2 antigen production yields harvested at 96, 168, and 216 h.p.i were normalized to the H_2-free control group. Experimental results represent the mean ± standard deviation (SD) in three individual virus sample repeats.
