# Supplementary material for: A strategy of novel molecular hydrogen-producing antioxidative auxiliary system improves virus production in cell bioreactor
Source: Sci Rep. 2024 Feb 19;14:4092. doi: 10.1038/s41598-024-54847-7 (PMC10876984; doi:10.1038/s41598-024-54847-7)
Supplement: Supplementary file 2 — Supplementary Table 1. [file 41598_2024_54847_MOESM2_ESM.pdf]

Supplementary Table 1. Performance of BHK-21 and PK-15 cells growth conditions in the BelloCell-500AP bioreactor and BEFV and PCV-2 production, respectively.

Medium(ml)	500			
Total surface area(cm^2)	15,600			
Virus and Cell lines	BEFV infected-BHK-21		PCV-2 infected-PK-15	
Multiplicity of infection (MOI)	0.01		0.008	
AAS supply	H_2-treated	H_2-free	H_2-treated	H_2-free
Total virus production ($TCID_{50}$)	1.4×10^{10}	3.7×10^{9}	1.3×10^{9}	5.1×10^{8}
Total virus production fold change to H_2-free	3.7		2.5	
